# The impact of the COVID-19 pandemic on antimicrobial prescribing at a specialist paediatric hospital: an observational study

**DOI:** 10.1093/jac/dkac009

**Published:** 2022-02-02

**Authors:** Emma Vestesson, John Booth, James Hatcher, Orlagh McGarrity, Neil J. Sebire, Adam Steventon, Carlos Suarez Alonso, Stephen Tomlin, Joseph F. Standing

**Affiliations:** 1 UCL Great Ormond Street Institute of Child Health, London, UK; 2 The Health Foundation, London, UK; 3 Great Ormond Street Hospital, London, UK; 4 NIHR GOSH BRC, London, UK

## Abstract

**Background:**

The COVID-19 pandemic has severely impacted healthcare delivery and there are growing concerns that the pandemic will accelerate antimicrobial resistance.

**Objectives:**

To evaluate the impact of the COVID-19 pandemic on antibiotic prescribing in a tertiary paediatric hospital in London, UK.

**Methods:**

Data on patient characteristics and antimicrobial administration for inpatients treated between 29 April 2019 and Sunday 28 March 2021 were extracted from the electronic health record (EHR). Interrupted time series analysis was used to evaluate antibiotic days of therapy (DOT) and the proportion of prescribed antibiotics from the WHO ‘Access’ class.

**Results:**

A total of 23 292 inpatient admissions were included. Prior to the pandemic there were an average 262 admissions per week compared with 212 during the pandemic period. Patient demographics were similar in the two periods but there was a shift in the specialities that patients had been admitted to. During the pandemic, there was a crude increase in antibiotic DOTs, from 801 weekly DOT before the pandemic to 846. The proportion of Access antibiotics decreased from 44% to 42%. However, after controlling for changes in patient characteristics, there was no evidence for the pandemic having an impact on antibiotic prescribing.

**Conclusions:**

The patient population in a specialist children’s hospital was affected by the COVID-19 pandemic, but after adjusting for these changes there was no evidence that antibiotic prescribing was significantly affected by the pandemic. This highlights both the value of routine, high-quality EHR data and importance of appropriate statistical methods that can adjust for underlying changes to populations when evaluating impacts of the pandemic on healthcare.

## Introduction

There is growing concern that the COVID-19 pandemic will accelerate antimicrobial resistance (AMR), an existing global health threat. High rates of antibiotic use in COVID-19 patients have been reported despite low rates of bacterial co-infections.^1^ But perhaps more relevant to children who are generally mildly affected by the disease, are behavioural and structural changes in society and in healthcare settings that might impact how antibiotics are being used. Factors such as increased pressure on healthcare workers, less opportunity for isolation of infectious patients and increased rates of empirical antimicrobial use for patients with respiratory symptoms could lead to increased antibiotic use; however, increased focus on hand hygiene in hospitals could reduce the spread of AMR and social distancing in society might lead to reductions in patients presenting at hospital with respiratory illnesses.^2^ Understanding the impact of the pandemic on antimicrobial use can inform antimicrobial stewardship (AMS) policies and responses to future pandemics.

This study aimed to evaluate the impact of the COVID-19 pandemic on antibiotic prescribing in a tertiary paediatric hospital in London, UK. Changes to the patient population were described and multivariable regression models were used to estimate the effect on antibiotic use.

## Patients and methods

### Setting

The UK implemented restrictions to limit the spread of COVID-19. A first nationwide lockdown was implemented on 23 March 2020 and schools had moved online on 20 March 2020. This was followed by a month-long second national lockdown in November 2020 and a third lockdown in January 2021.^3^

Great Ormond Street Hospital (GOSH) is a paediatric tertiary care hospital in London with an established AMS team.^4^ The AMS team comprises an antimicrobial pharmacist, an infectious disease consultant and a microbiology consultant and their work includes a weekly handover and ward rounds on four days of the week. AMS activities continued at the same level compared with pre-pandemic, however, the face-to-face stewardship rounds transitioned to a virtual format using the comprehensive electronic patient record. As part of a systems response to the pandemic, most complex paediatric inpatients in North Central London CCG were cared for at GOSH from April 2020, instead of their local hospital. Working patterns were also affected with more staff working remotely, being off sick or being deployed to other hospitals.

### Data

This study used routinely collected de-identified hospital data from inpatients at GOSH between 29 April 2019 and 28 March 2021 and who spent at least one night in hospital (ethics approval 17/LO/0008). Admissions data was linked to data on treatment speciality, surgical encounters and medication prescribing. Patients older than 25 years of age when admitted were excluded from the study (<1% of admissions) but no other exclusion criteria were applied.

Descriptive statistics of patient characteristics were derived from information recorded at admission (see Table S1, available as Supplementary data at *JAC* Online, for definitions).

Administration of any antimicrobial on a calendar day, regardless of the number of administrations, represented one day of therapy (DOT). The number of patient days, including the day of discharge, was used as the denominator to calculate DOTs per 1000 patient days. Antibiotics administered were then grouped into Access, Watch and Reserve groups using the classification developed by the WHO^5^ and the proportion of Access antibiotics was calculated. All analysis was carried out using R version 4.0.3 and the code is available (see Supplementary data section).^6^

### Interrupted time series model

Interrupted time series models were used to compare counts of weekly antibiotic DOTs and the percentage of Access antibiotics before the pandemic with the first year of the pandemic. The hypothesis was that the pandemic would cause an immediate and constant shift in antimicrobial consumption, commonly referred to as a level change with no lag. A negative binomial model with the number of patients days (logged) included as an offset was used for antibiotic DOTs and a binomial model was used to model the percentage of Access antibiotics. See Table S5 for full list of variables tested for inclusion in the model. Model residuals were checked for signs of autocorrelation and tested for using the Breusch–Godfrey test. The final model was selected using the Akaike Information Criterion.

## Results

There were 23 292 inpatient admissions (14 449 individual patients). There were 46 weeks included in the pre-COVID-19 period and 54 weeks in the COVID-19 period with each week contributing a minimum of 1450 patient days. During the pre-COVID-19 period 44% of antibiotic DOTs were from the Access group compared with 42% during the pandemic.

There was no meaningful difference in the median age between patients admitted before and during the pandemic, but those admitted in the COVID period were more likely to get at least one antibiotic, antiviral or antifungal during their stay (Table 1). A positive COVID-19 test was found for 134 admissions. Median weekly patient days by speciality before and during the pandemic and variation over time can be found in Table S3 and Figure S1.

**Table 1.** Patient-level characteristics before and during the COVID period

	In-patient admissions		
Characteristic	Pre-COVID-19	COVID-19	
	(*N* = 11 852)	(*N* = 11 440)	*P* value^a^
Age, years, median (IQR)	5.3 (1.8–10.7)	5.2 (1.4–11.2)	0.14
Male	6449 (55%)	6375 (56%)	0.30
Any theatre encounter	5577 (48%)	5023 (44%)	<0.001
Admission type			<0.001
Elective	9802 (84%)	8404 (74%)	
Emergency	690 (5.9%)	1185 (10%)	
Other	27 (0.2%)	46 (0.4%)	
Transfer	1203 (10%)	1798 (16%)	
Antibiotics during stay	5925 (51%)	6324 (55%)	<0.001
Antifungals during stay	945 (8.1%)	1153 (10%)	<0.001
Antivirals during stay	475 (4.1%)	605 (5.3%)	<0.001
Antiprotozoal during stay	99 (0.8%)	117 (1.0%)	0.20
Immunosuppressants during stay	413 (3.5%)	445 (3.9%)	0.14

a Wilcoxon rank sum test or Pearson’s Chi-squared test.

There was an increase in crude antibiotic and antiviral DOTs between the period before and during the pandemic (Table S4). Antibiotic DOTs by AWaRe group can be found in Table S5.

**Figure 1.**
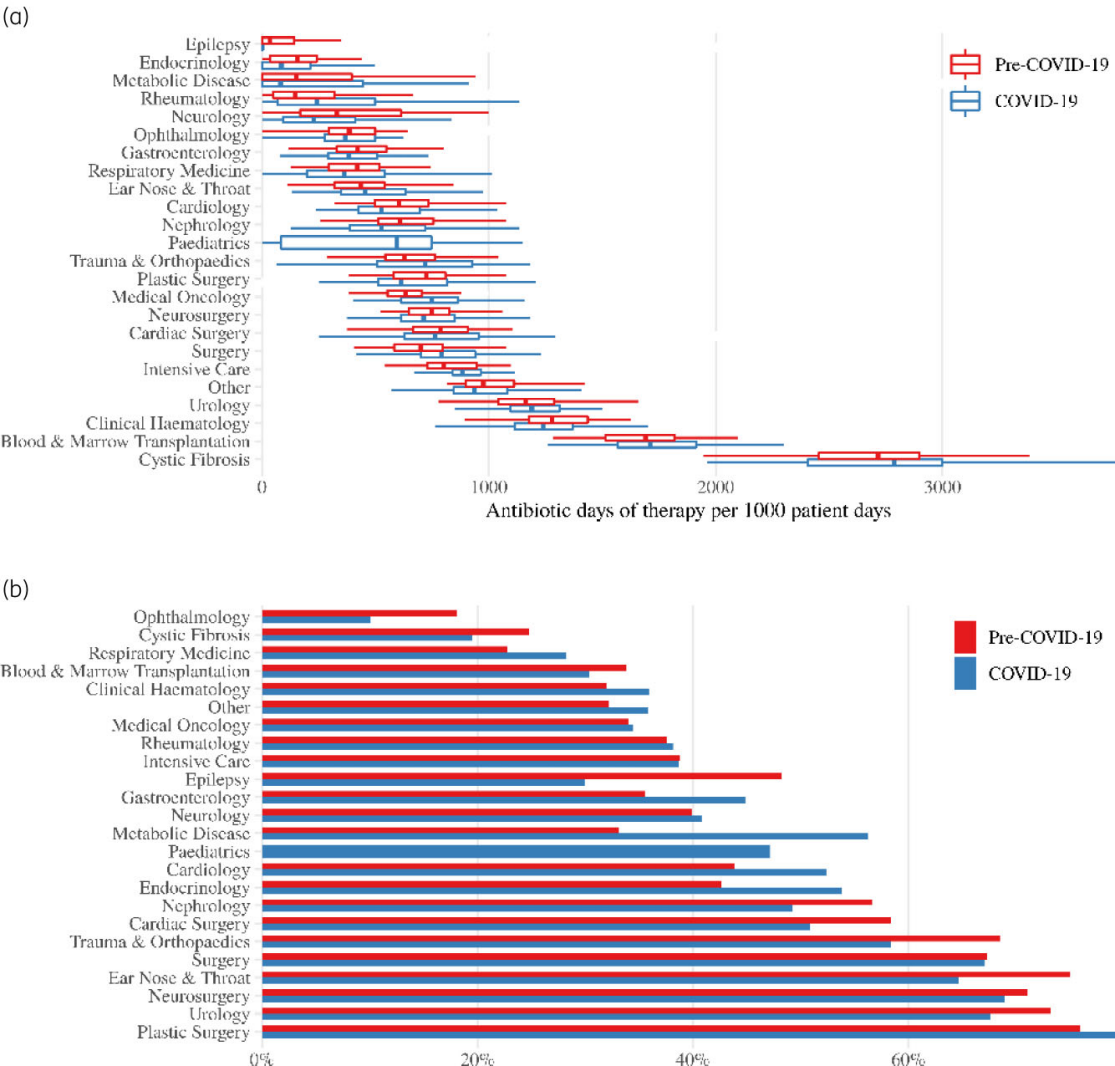
Antibiotic usage at GOSH before and during the COVID-19 pandemic. (a) Antibiotic DOT per 1000 bed days by speciality. Box shows the median and IQR, whiskers extend to lowest/largest values but no further than 1.5×lower/upper interquartile ranges. (b) Percentage of Access antibiotics. This figure appears in colour in the online version of *JAC* and in black and white in the print version of *JAC*.

There was considerable variation in antibiotic DOTs per 1000 patient days between specialities, but no speciality experienced a substantial change during the pandemic period (Figure 1a). There was substantial variation in the proportion of Access DOTs between specialities and the two specialities with highest antibiotic DOTs saw a decrease in the percent of Access DOTs (Figure 1b).

No statistically significant difference in antibiotic consumption could be detected for either antibiotic DOTs [incidence rate ratio 1.01 (95% CI: 0.95–1.08)] or percentage of Access antibiotics [odds ratio 0.83 (95% CI: 0.04–16.1)](Table S6). There was no evidence of autocorrelation (residual plots and autocorrelation tests can be found in Figures S2 and S3). Table S2 shows the variables included in the final model for both outcomes.

## Discussion

We found an increase in crude antibiotic DOTs per 1000 patient days but after adjusting for changes to the patient population using statistical modelling, there was no evidence of significant changes to antibiotic use during the first year of the pandemic.

The variation in changes in patient bed days between specialities explains most of the crude increase in antibiotic DOTs as there is substantial variation in antibiotic DOTs between specialities (Figure 1a). The large increases in intensive care patients during the COVID-19 pandemic are likely the result of transfers from other hospitals, as is the increase in number of cancer patients. These are patient groups with intrinsically greater use of antimicrobials. For specialities such as paediatric respiratory medicine, the reduction in bed days is likely a consequence of a decrease in demand due to behavioural changes during lockdown. The increase in the proportion of emergency admissions and decrease in surgeries will also have accounted for some of the crude differences.

Excessive antibiotic use in COVID-19 patients has been widely reported,^7^ suggesting a collapse in AMS. However, the impact of the pandemic on AMS activities is likely to be mixed. In a survey of hospitals and healthcare networks from June 2020, 65% of respondents thought that the pandemic had had a negative impact on routine AMS activities and 25% thought there were both positive and negative effects.^8^ This study shows the value of a dedicated AMS team.

The wider impact on antibiotic prescribing for all patients is mixed. Multiple studies have examined the pandemic impact on antibiotic prescribing in primary care in England and report a decrease in GP prescribing but an increase in dental prescribing.^9–11^ There was a 4.8% increase in total prescribing rate between 2019 and 2020 in secondary care in England but the patient population was vastly different to previous years.^12^ This study provides new insights on the pandemic impact on inpatient antimicrobial use in children whilst also considering the complex changes to patient population.

All data in this study was routinely collected and extracted from a digital database. This study demonstrates how hospital electronic health record (EHR) data can be used to evaluate important system changes and monitor antimicrobial use. The value of EHR data featured heavily in the UK’s five-year National Action Plan to tackle antimicrobial resistance.^13^ Despite this, a recent systematic review found that few antimicrobial use studies used solely digitally extracted data.^14^

A study strength is the use of a large routinely collected, comprehensive patient-level dataset. We used two different antibiotic use metrics that captured both volume changes and antibiotic type. The rich data and metric choice provide a more accurate overview of the changes to antimicrobial use and their appropriateness. Interrupted time series models were used which allowed us to control for the substantial changes in patient population.

This is a single-centre study from a specialist hospital with a small proportion of COVID-19 patients and no conclusions can be drawn about the overall effect of the pandemic on antimicrobial use in children in broader inpatient settings.

### Conclusions

Crude antimicrobial consumption increased during the COVID-19 pandemic but after adjusting for changes in case-mix, this association disappeared. This indicates that GOSH managed to continue to deliver vital healthcare treatments during the pandemic without compromising AMS practices.

## Supplementary Material

dkac009_Supplementary_Data

## Supplementary data

Tables S1 to S6 and Figure S1 to S3 are available as Supplementary data at *JAC* Online. The R code used for the analysis is available on https://github.com/emmavestesson/covid-ams.
