# Supplementary material for: The impact of the COVID-19 pandemic on antimicrobial prescribing at a specialist paediatric hospital: an observational study
Source: J Antimicrob Chemother. 2022 Feb 2;77(4):1185–8. doi: 10.1093/jac/dkac009 (PMC9383401; doi:10.1093/jac/dkac009)
Supplement: dkac009_Supplementary_Data [file dkac009_supplementary_data.docx]

# Supplementary data

#### Table S1 Definitions of key admission level variables and metrics

| Variable | Description |
| --- | --- |
| Age (years) | Age at admission in years |
| Male | Male sex recorded at admission |
| Any theatre encounter | Any theatre encounter during inpatient stay |
| Admission type | Type of admission recorded at admission |
| Antibiotics during stay | Antibiotics administered at least once during stay |
| Antifungals during stay | Antifungals administered at least once during stay |
| Antivirals during stay | Antivirals administered at least once during stay |
| Antiprotozoal during stay | Antiprotozoal administered at least once during stay |
| Immunosuppressants during stay | Immunosuppressants administered at least once during stay |
| Length of stay | Difference between the admission and discharge dates. Patients who were not discharged during the study period were censored on the 28 March 2021 which was then used to calculate length of stay. |
| Admission speciality | Treatment function codes were used to identify which specialised service that patients were admitted to. Included in the ‘other’ category if missing. |
|  |  |

#### Table S2 Variables included or tested for inclusion in models

| Variable | Description | Antibiotic DOTs | Percent from Access group |
| --- | --- | --- | --- |
| Covid | 0 if before 16 March 2020, 1 otherwise | Yes | Yes |
| Week since start | Integer indicating number of weeks since start of study | Yes | Yes |
| Month | Calendar month based on earliest date in week | No | No |
| Harmonic term – yearly pattern | sin(2*pi*time/52) + cos(2*pi*time/52) | No | Yes |
| Harmonic term – 6 month pattern | sin(2*pi*2*time/52) + cos(2*pi*2*time/52) | Yes | No |
| Harmonic term – 4 monthly pattern | sin(2*pi*3*time/52) + cos(2*pi*3*time/52) | No | Yes |
| Total working days | Total working days per week | Yes | Yes |
| Theatre encounters | Theatre encounters per 1000 patient days | Yes | No |
| Age less than 1 year | Patient days for patients under the age of 1 per 1000 patient days | Yes | Yes |
| Cystic fibrosis | Patient days for patients admitted under cystic fibrosis speciality per 1000 patient days | Yes | No |
| Clinical haematology | Patient days for patients admitted under clinical haematology speciality per 1000 patient days | Yes | No |
| Blood and marrow transplantation | Patient days for patients admitted under blood and marrow transplantation speciality per 1000 patient days | Yes | No |
| Immunosuppressant drugs | Patient days for patients getting immunosuppressant drugs per 1000 patient days | Yes | No |

#### Table S3 Weekly patient days by speciality before and during the pandemic

|  | Pre-COVID-19^a^, N = 46 | COVID-19^a^, N = 54 | p-value^b^ |
| --- | --- | --- | --- |
| Blood And Marrow Transplantation | 86 (78, 96) | 104 (90, 120) | <0.001 |
| Other | 196 (184, 241) | 150 (135, 160) | <0.001 |
| Cardiac Surgery | 79 (68, 90) | 61 (44, 74) | <0.001 |
| Cardiology | 64 (53, 77) | 77 (69, 90) | <0.001 |
| Clinical Haematology | 106 (82, 121) | 98 (85, 113) | >0.9 |
| Cystic Fibrosis | 30 (26, 34) | 31 (27, 35) | 0.8 |
| Ear Nose And Throat | 64 (54, 74) | 46 (26, 61) | <0.001 |
| Endocrinology | 52 (44, 61) | 33 (22, 43) | <0.001 |
| Epilepsy | 24 (18, 29) | 10 (6, 14) | <0.001 |
| Gastroenterology | 74 (60, 83) | 60 (49, 72) | <0.001 |
| Intensive Care | 308 (283, 380) | 421 (372, 456) | <0.001 |
| Medical Oncology | 123 (106, 130) | 132 (114, 156) | 0.021 |
| Metabolic Disease | 28 (18, 31) | 14 (7, 17) | <0.001 |
| Nephrology | 70 (60, 82) | 62 (52, 70) | 0.001 |
| Neurology | 42 (32, 49) | 37 (29, 43) | 0.017 |
| Neurosurgery | 93 (82, 108) | 87 (74, 100) | 0.067 |
| Ophthalmology | 10 (6, 13) | 9 (6, 13) | 0.9 |
| Plastic Surgery | 58 (43, 69) | 37 (17, 52) | <0.001 |
| Respiratory Medicine | 124 (117, 132) | 90 (70, 100) | <0.001 |
| Rheumatology | 27 (21, 33) | 23 (17, 31) | 0.2 |
| Surgery | 98 (88, 114) | 95 (80, 116) | 0.4 |
| Trauma And Orthopaedics | 76 (66, 86) | 38 (20, 58) | <0.001 |
| Urology | 73 (58, 79) | 58 (43, 80) | 0.045 |
| Paediatrics | 0 (0, 0) | 7 (0, 66) | <0.001 |
| ^a^Median (IQR) | | | |
| ^b^Wilcoxon rank sum test | | | |

####

Table S4: Change in DOTs per 1000 patient days before and during the pandemic

|  | Pre-COVID-19^a^, N = 320 | COVID-19^a^, N = 377 | p-value^b^ |
| --- | --- | --- | --- |
| Antibiotic DOT per 1000 patient days | 801 (764, 854) | 846 (805, 903) | <0.001 |
| Antifungal DOT per 1000 patient days | 186 (169, 205) | 192 (175, 211) | 0.016 |
| Antiviral DOT per 1000 patient days | 65 (54, 76) | 78 (67, 90) | <0.001 |
| Antiprotozoal DOT per 1000 patient days | 4 (0, 7) | 4 (0, 5) | 0.043 |
|  |  |  |  |
| ^a^Median (IQR) | ^b^Wilcoxon rank sum test |  |  |

Table S5: Weekly antibiotic days of therapy for the AWaRe groups

|  |  | Pre-COVID-19^a^, N = 46 | COVID-19^a^, N = 54 | p-value^b^ |
| --- | --- | --- | --- | --- |
|  | Access | 369 (351, 387) | 377 (358, 396) | 0.15 |
|  | Watch | 417 (390, 442) | 463 (439, 476) | <0.001 |
|  | Reserve | 27 (22, 31) | 24 (17, 29) | 0.024 |
| ^a^Median (IQR) | | | | |
| ^b^Wilcoxon rank sum test | | | | |

Table S6: Interrupted time series model results

|  | Antibiotic DOTs | | | | | | Percent from Access group | | | | | |
| --- | --- | --- | --- | --- | --- | --- | --- | --- | --- | --- | --- | --- |
|  | Unadjusted | | | Adjusted | | | Unadjusted | | | Adjusted | | |
|  | IRR^a^ | 95% CI^a^ | p-value | IRR^a^ | 95% CI^a^ | p-value | OR^a^ | 95% CI^a^ | p-value | OR^a^ | 95% CI^a^ | p-value |
| COVID-19 period | 1.08 | 1.04, 1.12 | <0.001 | 1.01 | 0.95, 1.08 | 0.7 | 0.93 | 0.19, 4.50 | >0.9 | 0.83 | 0.04, 16.1 | >0.9 |
| No. Obs. | 100 |  |  | 100 |  |  | 100 |  |  | 100 |  |  |
| AIC | 1,161 |  |  | 1,159 |  |  | 119 |  |  | 133 |  |  |
| ^a^IRR = Incidence Rate Ratio, CI = Confidence Interval, OR = Odds Ratio | | | | | | | | | | | | |

Figure S1: Heatmap of minimum-maximum normalised weekly patient days by speciality (normalised within speciality)
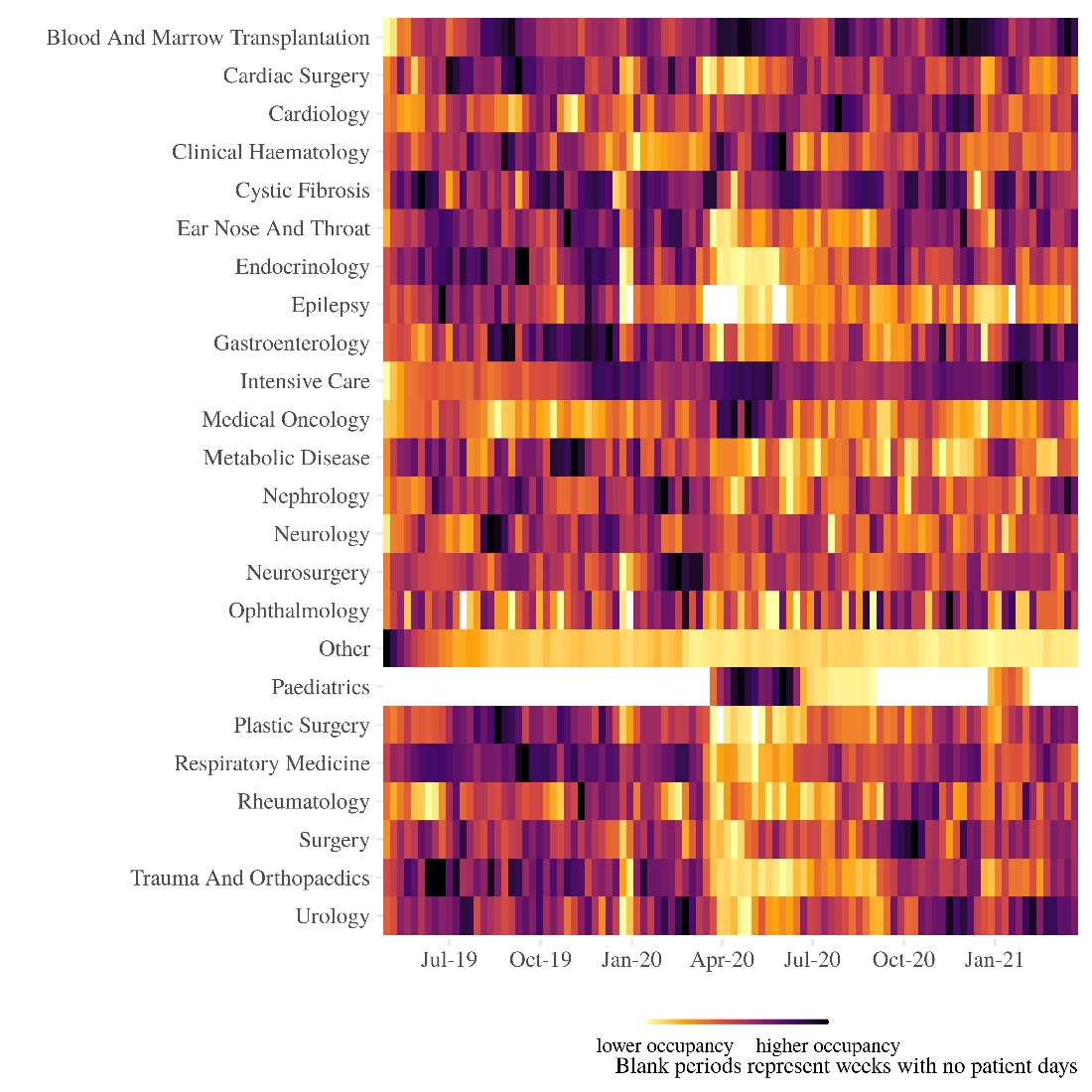


#### Figure S2 Residuals over time - antibiotic DOT


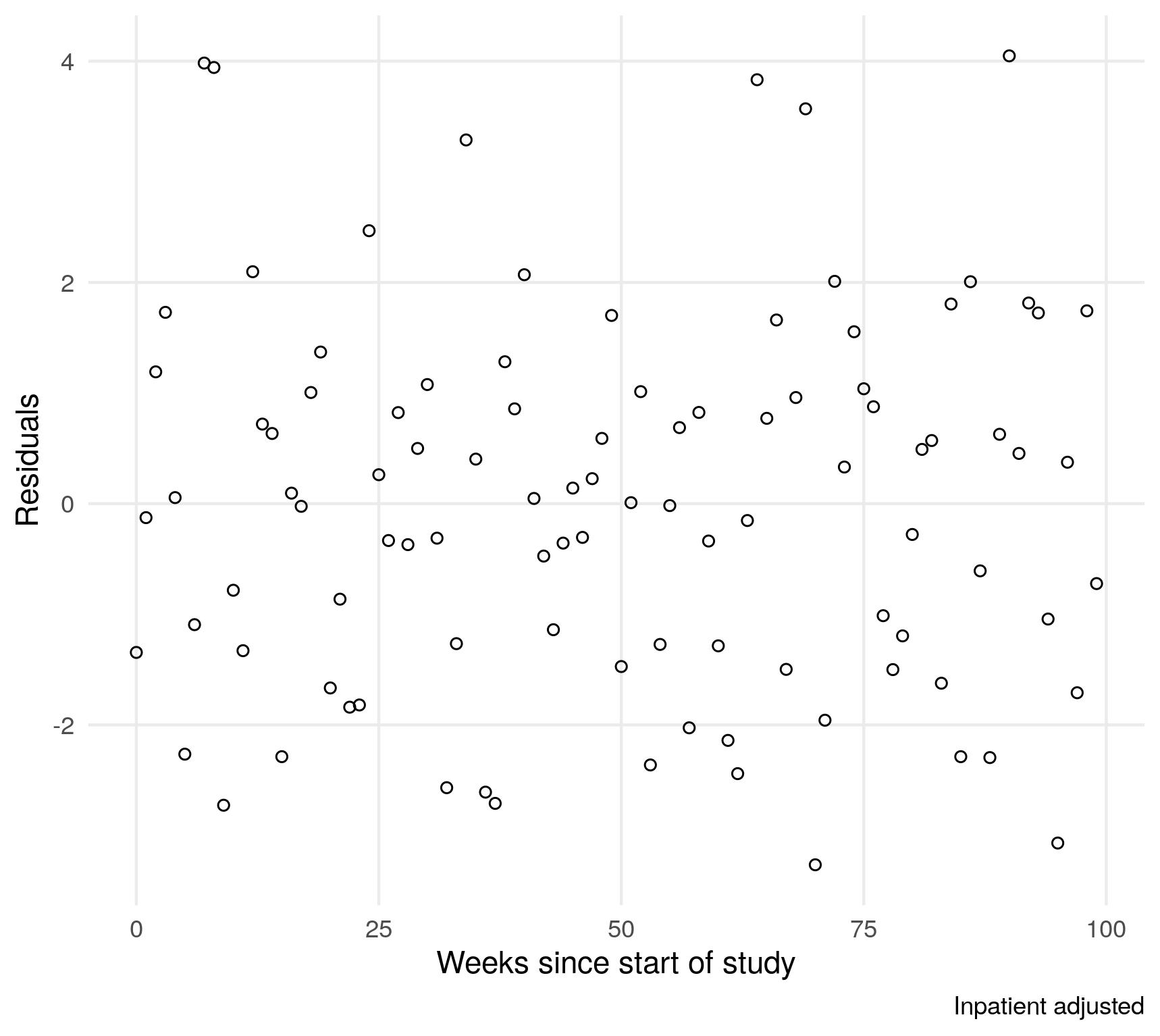


Residuals vs time (DOTs)

DOTs: Breusch-Godfrey test, p-value=0.1532

#### Figure S3 Residuals over time - percent from Access group


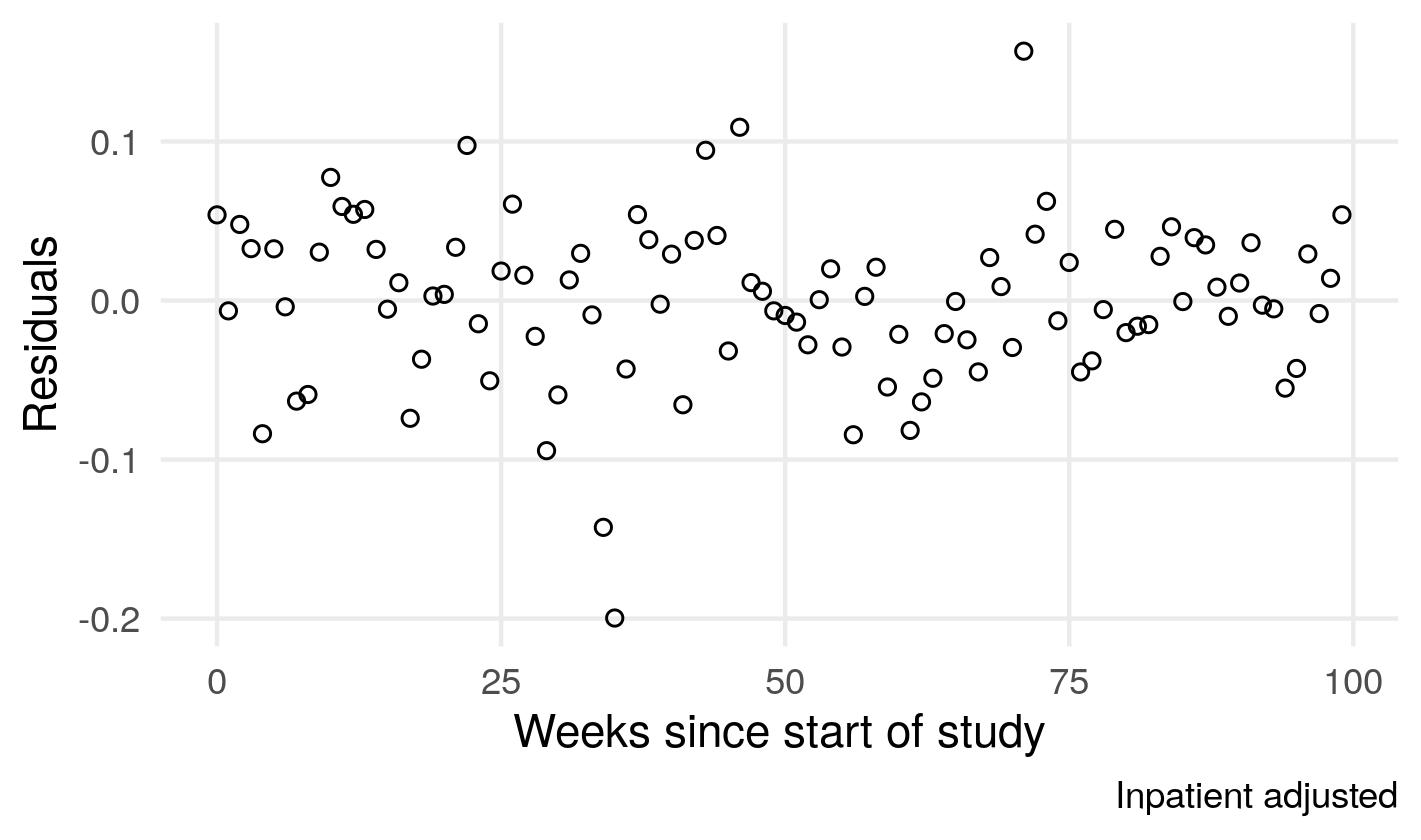


Residuals vs time (Access)

Access: Breusch-Godfrey test, p-value=0.05584
